# Treatment of Cognitive Impairment in Multiple Sclerosis

**DOI:** 10.1155/2006/545860

**Published:** 2006-05-11

**Authors:** Susan H. Pierson, Nathan Griffith

**Affiliations:** ^1^Department of NeurologyDrake Center and University of Cincinnati151 West Galbraith RoadCincinnatiOH 45216USA; ^2^Department of PsychologyUniversity of CincinnatiCincinnatiOH 45219USA

**Keywords:** Multiple sclerosis, cognitive dysfunction, fatigue, employment, disease characteristics, working memory, executive dysfunction, visuospatial perception, depression, pharmacologic management, non-pharmacologic treatments

## Abstract

Cognitive impairment in multiple sclerosis is an increasingly recognized entity. This article reviews the cognitive impairment of multiple sclerosis, its prevalence, its relationship to different types of multiple sclerosis, and its contribution to long-term functional prognosis. The discussion also focuses on the key elements of cognitive dysfunction in multiple sclerosis which distinguish it from other forms of cognitive impairment. Therapeutic interventions potentially effective for the cognitive impairment of multiple sclerosis are reviewed including the effects of disease modifying therapies and the use of physical and cognitive interventions.

## 1. Introduction

The effect of multiple sclerosis (MS) on cognition has gained increasing recognition as potentially the most disabling symptom of the disease. Long under-recognized and thought to affect only a minority of persons with MS, cognitive impairment has moved steadily to the forefront through the groundbreaking work of Stephen Rao [95–99,101]. Outcome measures in clinical trials and practice have begun to emerge to address this particular aspect of MS. The Multiple Sclerosis Functional Composite (MSFC) was a scale created in 1994 by the National Multiple Sclerosis Society (NMSS) via a Clinical Outcomes Assessment Task Force [45] and is now widely in use in MS clinics and trials. The lone cognitive measure on the MSFC is a modification of the Paced Auditory Serial Addition Test (PASAT) [58].

As the role of magnetic resonance imaging (MRI) and its use to monitor disease progression has advanced, atrophy and lesion load have been debated, yet increasingly accepted, as surrogate measures of disease progression and cognitive decline [41,106]. Benedict et al. [21] correlated personality style, neuropsychological performance and major depression with the presence of atrophy and lesion burden on MRI. Atrophy, in particular, was predictive of euphoria and disinhibition. Both lesion burden and atrophy predicted major depression in this study. Research at the University of Buffalo is focused on the relationship between regional brain atrophy, cognitive, and emotional impairment [21].

Undoubtedly, the physical and emotional symptoms of MS can be extremely distressing, but cognitive impairment may be the most devastating symptom for some patients and their families [59]. The social costs of cognitive impairment, especially when coupled with fatigue or depression, may be enormous, and stem, in large part, from the progressive inability of the patient to perform competitively in employment and social situations. Affective disorders exist concurrently, or arise as a consequence of socio-economic concerns, and make the symptom constellation more complex.

ISSN 0953-4180/06/$17.00 © 2006 – IOS Press and the authors. All rights reserved

Cognitive decline has now been shown to be the major reason for disability and withdrawal from the work force for persons with MS [98].

Cognitive impairment can profoundly impair family life and social relationships. Cognitively impaired patients with MS report loss of self-esteem, sexual dysfunction, and divorce more frequently than non-impaired MS patients with similar levels of physical disability [98]. Cognitive impairment can also affect relationships with friends and colleagues. Cognitive impairment may dictate that a person with MS stop driving or traveling independently, resulting in restricted mobility and a loss of independence. Restricted mobility may, in turn, complicate the logistics of planning and attending social activities. Furthermore, patients may have trouble remembering scheduled activities, participating in fast-paced conversations or activities, or may lose interest in previously enjoyed activities. Research has shown that cognitively impaired patients with MS are significantly more likely to demonstrate impaired social functioning than healthy controls. In addition, they are less likely to be engaged in social activities and more likely to need assistance with personal care, household management, and activities of daily living [10,61,98]. In addition, cognitively impaired persons with MS are more likely to manifest psychopathology (e.g. affective disorders, anxiety disorders) than cognitively intact patients with comparable physical disability or healthy controls.

Cognitive impairment also affects the employment status of persons with MS [18,98–101]. A study sponsored by the NMSS (National Multiple Sclerosis Society) found that cognitive factors and physical fatigue are the two biggest barriers to employment among MS patients [6]. Rao and colleagues reported that 50 to 80% of MS patients are unemployed within 10 years of disease onset [98]. This high rate of loss of employment does not seem to be related to degree of physical or neurological disability; selected demographic variables and degree of physical disability accounted for less than 14% of the variability [72]. Driving ability, an important skill in terms of maintaining employment, is affected by cognitive impairment. In a retrospective study of driving records, cognitively impaired MS patients had significantly more motor vehicle collisions than cognitively intact MS patients or healthy controls [108].

Cognitive impairment may also have indirect effects on employment status; Cognitive impairment hinders a patient's ability to benefit from cognitive or vocational rehabilitation [71]. Clemmons et al. [33] suggested that the sparing of verbal abilities, typical in the cognitively impaired person with MS, might divert attention from other areas of cognitive weakness. MS patients may tend to experience and communicate a desire for, increased intellectual challenges at work. This desire for challenge may be a function of high levels of educational status and occupational attainment due to the typical "mid-career" onset of the disease. The combination of loss of abstraction abilities, cognitive efficiency, and the desire for increased responsibility or task complexity makes a person with MS particularly susceptible to becoming overwhelmed with responsibilities or complicated duties at work. Although it is clear that MS patients frequently experience work-related difficulties and job transition, the process of vocational counseling and job placement for persons with MS has been understudied [33].

A review of the literature by Pompeii et al. [91] revealed that while cognitive impairment and physical disability were a definite hindrance to working, there were other complicating factors including level of education, socio-economic difficulties, job characteristics, and disease symptoms such as fatigue. Physical and cognitive measures alone were not predictive of patients who were or were not able to work [91,108].

Multiple sclerosis is a progressive, insidious disease that affects not only physical capacity, but every other aspect of a patient's life, including independence of personal care, fulfillment of social and family roles, sexual functioning, cognitive functioning, employment status, and economic security. A patient's perception of their own degree of overall health and physical, psychological, and social functioning is known as quality of life (QOL). Health-related quality of life (HRQOL) is a similar construct, assessed via a patient's perspective on the impact and treatment of their disorder on their quality of life. Unlike other clinical measures of disease-related variables, HRQOL is not measured from the perspective of physicians. HRQOL is growing in popularity among investigators who work with patients with medical and neurological disorders, including MS. It is recognized that most aspects of HRQOL are weakly correlated with traditional measures of neurologic impairment; this suggests that HRQOL, which can be assessed alongside more traditional measures of impairment, contributes unique information about a patient's status [45]. MS patients may be more concerned with quality of life (e.g. mental health, emotional distress, vitality) than with physical disability [105]. There is evidence that MS patients views differ from their treating physicians on the importance placed on quality of life [105].

Neuropsychological assessment can be particularly useful in helping a person with MS find and maintain appropriate employment. The results of a neuropsychological assessment can inform many future choices a person with MS may need to make. Neuropsychologists may offer recommendations designed to maximize a patient's ability to succeed in a work setting (e.g. appropriate employment settings to fit the patient's strengths, appropriate external aids that will support job performance). Furthermore, interpretation of a neuropsychological assessment can sensitize the patient to areas of challenge and alert employers to the need to modify job duties to match the strengths and limitations of an employee with MS.

## 2. Characteristics of cognitive impairment in MS

The prevalence of MS in the United States is between 250,000 and 300,000 [11]. The prevalence of cognitive impairment in MS is estimated to be present in 50 to 75% of persons with MS [98]. Cognitive impairment may be an early manifestation of the disease in a small proportion of patients, but tends to develop late in the course of the disease in the majority of affected cases. Cognitive impairment tends to be most severe in patients with disease of long duration and severe physical disability [2]. Amato et al. [9] demonstrated cognitive impairment in 56% of his cohort who had had disease of at least 10 years duration. He identified clinical variables which were predictive of cognitive decline, including progressive disease course, level of physical disability, and increasing age. The transition from the relapsing remitting to the relapsing remitting secondarily progressive phase of the disease may represent the critical juncture at which cognitive decline develops [34].

The common deficits in cognitive function which appear in MS owe, in large part, to the subcortical nature of injury inflicted by plaque and inflammation. These cognitive deficits may be linked to the disconnection of brain regions from one another, as irreversible axonal loss in the subcortex renders crossing fibers and connection fasiculi ineffective in relaying messages and information from one brain region to another [118]. Nearly uniformly, the patients present with some pattern of subcortical impairment. Additionally, MRI and autopsy studies have shown sclerotic plaques to be present in the gray matter as well as the subcortical white matter, and these injuries also are likely to play a role in the process of cognitive decline [54,65].

Reviews by researchers [53,95,98,122] have summarized the most frequently affected neurocognitive domains in MS. They are: a) speed of information processing; b) sustained attention/working memory; c) learning and memory; d) executive functions (e.g. verbal fluency, planning, novel problem solving); and e) visuospatial abilities (e.g. visual-perceptual, visuospatial, and constructional abilities).

A functional MRI study showed that during working memory tasks, patients with MS exhibit a pattern of activation beyond the typical components of working memory circuitry [123]. While these changes in activation may be compensatory in nature, they may also reflect passive redirection of neural activity, failed inhibition due to the MS lesions, axonal transactions, and diffuse microscopic changes associated with the disease. The cortical domains of cognition (e.g. language, praxis, neglect) are generally unaffected in MS, though slowed speed of cognitive processing may make word-finding difficult and fluency less automatic. Impairment in sustained and divided attention, new learning, executive function, and visuospatial skills are more typical of the pattern of cognitive decline seen in MS [2]. Overall IQ does not tend to drop and encoding of memory appears intact. Retrieval, on the other hand, is impaired. Both cueing and context can benefit retrieval in persons with MS [82]. Impaired conceptual reasoning and deficits in auditory and visual span also occur, albeit less frequently than the other areas noted above.

Visuospatial perception is more commonly impaired in MS than was previously thought. How the pathophysiology of MS relates to this deficit is unclear, as primary visual association cortices are generally spared. It is possible visuospatial deficits arise as a result of primary visual impairment (e.g. optic neuritis or opthalmoplegia). Visuospatial difficulties may also arise as a result of higher order deficits in complex visual processing [98]. Persons with primary progressive MS seem to fair worse cognitively than those persons with relapsing remitting secondarily progressive MS. There was also an insignificant trend toward poor cognitive performance by persons with MS who were medicated for depression [98].

Short-term memory or immediate recall seems to remain preserved in MS [101] but these patients demonstrate significant impairments in verbal and nonverbal acquisition of new material or encoding of information. Retrieval, as previously mentioned, is consistently impaired. It is a condition made worse by the often concurrent slowing of processing speed [75]. "Accelerated forgetting" also occurs as the disease progresses.

Demyelinating lesions in the parahippocampal gyrus and the hippocampus may be singularly responsible for the memory impairment in MS [86]. The slowing of attention-demanding information processing reflects the loss of more complex cognitive skills which deteriorate in general. Patients with MS suffer from compromised central processing as well as from deficits in divided and sustained attention [36].

Foong et al. [49] described executive function as the abilities required for decision-making, planning, developing and initiating novel sequences of action, error correction, and overcoming habitual responses. In terms of deficits in executive function, MS patients may struggle to complete tasks, start new tasks while previous tasks are unfinished, have problems adapting to new or complex environments, or may be unable to solve problems which are especially novel or complex [59]. In addition, MS patients may be differentially aware of their cognitive deficits. Patients typically complain of increased mental slowness, distractibility [42] and memory problems, but are often unaware of deficits in executive and visuospatial functioning.

Impairments in insight and abstract reasoning lead to the great strain caregivers feel in their relationship with the person with MS [56]. Executive functions are poorly localizable but there is ample evidence to suggest that MRI abnormalities correlate with the severity and characteristics of cognitive abnormalities in MS [14]. The functions of the anterior system (e.g. executive skills, abstract reasoning, judgment, and insight) are interconnected with other brain functions and are dependent on intact infrastructure to relay information and provide continuous feedback so that the anterior systems may function optimally.

Rao and colleagues [98] have characterized the typical pattern of cognitive deficits observed in MS as similar to subcortical dementia, in that deficits in sustained attention and concentration, verbal learning, visuospatial learning, and semantic retrieval are most common. As described by Albert et al. [8], patients with subcortical dementia “forget to remember” and demonstrate significant impairment in abstraction, fluency, cognitive flexibility, personality style (apathy and abulia), and speed of cognitive processing.

## 3. Timing and onset of cognitive impairment in MS

Once cognitive impairment develops, it is unlikely to reverse itself or remit. MRI measures of atrophy, thought to be a marker of secondary progression, have been shown to correlate with cognitive decline in MS. In a ten-year follow-up study of MS patients with cognitive decline, Amato et al. [9] were able to identify those factors which predicted severity of cognitive deterioration. Progressive disease, ample physical disability, and an aggressive disease course were most likely to contribute to cognitive impairment [9]. Yet, there is a subset of patients in whom cognitive decline is unlikely. This subset of patients, studied by Kujala et al. [64], performed as well on three-year follow-up as they did at baseline on a number of neuropsychological measures. However, those patients with even very mild cognitive decline at the outset of the study demonstrated an incipient worsening over the 3 year period. In their study, simple attentional tasks (digit span) and naming tasks, expected to remain stable, worsened over time. His group found no correlation between disease duration, physical disability, or disease course and cognitive disorder, in contrast with the other studies previously mentioned [64].

Further insight into the cognitive impairment of MS is provided by a longitudinal study in which disease evolution, cognitive impairment, and MRI lesion burden were monitored over time, with a mean 8.5 year follow-up. Increased MRI lesion burden did not correlate in this study with cognitive impairment. The authors postulated that cognitive impairment could be related to disease peculiarity and not to time course [89].

## 4. Concurrent disorders: Fatigue and depression

The concurrent existence of affective disorders and fatigue in so many patients with MS confounds the clinical picture and can make the cognitive impairment look worse than it truly is. A causal relationship between fatigue and cognitive decline has not been clearly established [62,66,85,109]. However, patients experiencing fatigue seem to concurrently report or self-perceive impairments in cognitive performance. In a study which compared the effects of 4-aminodipyridine and fluoxetine in fatigue, baseline fatigue scores correlated with depression and cognitive test scores [104]. Little scientific evidence supports the theories behind fatigue in MS. The pathophysiology of fatigue in MS is thought to result from some form of neuromodulation by products of the inflammatory autoimmune process, or to white matter injury which leads to deficits in the ability to sustain attention [110]. Difficulty in measuring fatigue has impeded studies of its characteristics, mechanisms, and therapeutics. Schwid et al. [110] suggest that all aspects of fatigue, from motor weakness to cognitive fatigue, should be measured through more quantifiable means. That limitation translates to a persistent and inadequate understanding of the relationship between fatigue and cognitive impairment.

Cognitive impairment may be compounded by the effects of other symptoms seen in MS, such as spasticity and pain, sleep impairment, depression, and immobility. Depression is so common in MS (prevalence of 37–54%), it is reasonable to assume it has a negative impact on cognitive performance. However, that link is not clearly established. There is an inverse relationship between cognitive impairment and depression. Cognitively intact patients are more likely to complain of memory problems yet test within normal ranges on neuropsychological measures [94]. The reverse is also true. Patients with significant cognitive impairment demonstrate diminished insight and thereby less depression [25]. Memory function and emotional changes were found to be linked in a study in which subjective complaints of memory problems were more common in the depressed patient with MS. However, impaired memory task performance was not correlated with those subjective complaints, nor with depressive symptoms, or with degree of physical disability [70].

A significant degree of depression must exist in order to create cognitive impairment (the pseudo-dementia of depression) and the cognitive impairment seen as a result of depression does not affect all cognitive domains equally [14]. Memory disorders in MS were found to coexist with the following: impairment in other cognitive domains; lower socioeconomic class; chronic progressive MS; and use of anti-anxiety medication. They did not coexist with severity or duration of disability, nor depression in a study by Minden et al. [79].

## 5. Outcome measures/surrogate markers of cognitive impairment and response to treatment

In the last three decades, considerable improvement has been made in the methods used by neurologists and other treating clinicians to assess functional impairment and outcome of treatment. Brief MS-specific neuropsychological batteries allow neuropsychologists to quickly assess cognitive functioning by focusing on the cognitive domains most frequently affected in MS. Clinicians typically assess the negative impact of MS on a patient's well-being by using a combination of instruments that provide information pursuant to a systematic classification system, the World Health Organization's (WHO) International Classification of Functioning, Disability, and Health (The ICIDH-2) [3]. Like its predecessor, the International Classification of Impairments, Disabilities, and Handicaps [4], it establishes a worldwide system for categorizing the impact of disease on three levels: the body level (previously called "impairment"); the personal level (previously called "disability"); and the societal level (previously called "handicap").

Clinical assessment of functional impairment in MS is difficult because of the large number of symptoms that may negatively impact daily functioning (e.g. loss of mobility, sensory-perceptual impairment such as temporary vision loss, cognitive impairment). In addition, the unpredictable manifestation, remission, and relapse of symptoms and the variable course of MS complicate the assessment of disease status. Physicians and others have realized that traditional neurological measures, such as the Kurtzke's Estimated Disability Status Scale (EDSS) [35,69], are not sensitive to important sequelae of MS-related neurologic disability, such as emotional distress and cognitive impairment. Mental status exams, such as the Mini-Mental Status Examination [19,48,98] are not sufficiently sensitive to detect cognitive impairment in MS. Moreover, bedside examinations of cognitive functioning and clinical interviews are insufficiently sensitive to detect cognitive impairment in MS, no matter how skilled the physician [43,87]. Indeed, cognitive functions that are typically assessed at the bedside, such as orientation, simple attention span, language, and immediate recall, are typically preserved in MS [98].

The modified PASAT has become an important cognitive test because it is widely used in MS research and clinical assessment; in fact, it is frequently used as the only indicator of cognitive functioning. The PASAT is very sensitive to impaired processing speed among MS patients. However, the PASAT is an extraordinarily difficult and frustrating test for both examinees and examiners. Also, it is unclear if the PASAT primarily measures processing speed, working memory, sustained attention, or calculation ability [46].

The most accurate method of assessing a wide range of cognitive functions is to administer a neuropsychological assessment. The results of a neuropsychological assessment provide a profile of the patient's cognitive strengths and weaknesses. The pattern of neuropsychological deficits often observed in MS patients is heterogeneous and may be subtle; therefore, most neuropsychologists administer a relatively thorough battery of tests when testing a patient with MS. However, there are barriers to administration of a comprehensive neuropsychological evaluation, including time constraints, cost, and limitations of patients' stamina and fine motor dexterity.

Several screening tests for MS-related cognitive impairment have been developed, including the Screening Examination for Cognitive Impairment [18], the Basso Screening Battery [17], and the NP Screening Battery [50]. These various screening tests take 15 to 45 minutes to complete. In contrast, a few research teams have proposed guidelines for developing a comprehensive neuropsychological assessment battery for use in assessing cognitive impairment in MS [87,88, 98]. Benedict et al. [22] and others have criticized the above screening batteries as being under-inclusive and too brief to adequately screen for cognitive impairment in MS; on the other hand, they have also criticized the above proposed comprehensive batteries for being too lengthy and overly thorough.

Several 'brief' neuropsychological assessment batteries have been proposed as a compromise between short screenings and comprehensive batteries. Two such batteries that have been frequently used are the Minimal Assessment of Cognitive Function in Multiple Sclerosis (MACFIMS) [22] and the Brief Neuropsychological Battery (BFNP) [98]. The MACFIMS was developed by an expert panel convened by the Consortium of Multiple Sclerosis Centers. The MACFIMS is a 90-minute battery comprised of seven tests. The battery was designed to measure 5 cognitive domains considered especially relevant to MS. They are (in order of rated importance): processing speed/working memory; learning and memory; executive functions; visual-spatial processing; and language/verbal skills [22]. The MACFIMS also includes the North American Adult Reading Test [24] and the Chicago Multiscale Depression Inventory (CMDI) [83] as ancillary tests. Rao and colleagues developed the BFNP, which consists of 4 tests: the Selective Reminding Test [26]; 7/24 Spatial Recall Test [16,97]; PASAT [58]; and a word list generation task.

## 6. Measuring quality of life in MS

One of the first disease-specific HRQOL measures developed for MS was the Minimal Record of Disability (MRD) [5]. The MRD consists of the EDSS, the Functional Systems Scale (FSS), the Incapacity Status Scale (ISS), and the Environmental Status Scale (ESS). In terms of the ICIDH-2, the EDSS and FSS measure the impact of MS at the body-level (impairment), the ISS is a person-level (disability) measure, and the ESS is a societal-level (degree of handicap) measure. However, the MRD was never widely adopted in MS care [45].

Three MS-specific standardized measures of HRQOL have recently been developed and endorsed by the NMSS: The Multiple Sclerosis Quality of Life-54 (MSQOL-54) [119,120]; the Functional Assessment of Multiple Sclerosis (FAMS) [29]; and the Multiple Sclerosis Quality of Life Inventory (MSQLI) [102]. Each of these measures was developed by supplementing well-validated generic HRQOL measures with MS-specific content. Some researchers regard these "hybrid" measures to be optimal disease-specific HRQOL measures. Each of the following MS-specific HRQOL measures was designed to be administered to either individual patients or groups of patients, which may provide researchers and clinicians with flexibility in assessment.

### *6.1. MSQOL-54*

The MSQOL-54 consists of the 36-item short form health survey (SF-36) and 18 additional MS-specific items. The MSQOL-54 has 12 subscales. In a recent review of QOL measures in MS, Fischer et al. [42] stated that the MSQOL-54 is limited in its assessment of concerns related to visual function and incontinence.

### *6.2. FAMS*

The FAMS consists of a 28-item measure of HRQOL developed for use among cancer patients, the Functional Assessment of Cancer Therapy-General (FACT-G) [30] and additional MS-specific items. The FAMS has 6 MS-relevant subscales: Mobility, Symptoms, Emotional Well-being, General Contentment, Thinking/Fatigue, and Family/Social Well-being. Fischer et al. [45] asserted that the FAMS over-represents the psychosocial sequelae of MS, while under-representing concerns related to visual and sexual functioning and incontinence.

### *6.3. MSQLI*

The Multiple Sclerosis Quality of Life Inventory (MSQLI) differs from the MSQOL-54 and the FAMS in that the test developers added established symptom-specific scales, rather than selected symptom-specific items, to an existing, generic HRQOL measure. Like the MSQOL-54, the generic HRQOL measure contained within the MSQLI is the SF-36. The MSQLI is a comprehensive assessment of HRQOL, which suggests superior content validity over other MS -specific HRQOL measures. However, the advantage of breadth has an associated drawback: the MSQLI is about twice as long as the above MS-specific HRQOL measures.

## 7. Prevention of worsening: Pharmacologic and medical management of cognitive impairment in MS

All of the immuno-modulating agents available in the US have shown benefit for persons with MS in the form of reduced numbers of attacks and decreased lesion load by MRI. Evidence for benefit from immunomodulating agents in preventing cognitive impairment is less robust, as none of the original studies, other than the pivotal trial with Avonex® versus placebo, incorporated cognitive evaluations as part of the outcome measure. There were 276 patients in the initial Avonex® trial and 166 available for two-year follow-up. A comprehensive neuropsychological battery was administered and included measures for information processing speed and memory, visuospatial abilities, executive function, verbal abilities, and attention span. Overall neuropsychological outcome improved in 53.2% of Avonex® treated patients compared with controls, while 29% remained stable. Despite treatment with Avonex®, 17.7% did progress in their cognitive decline. These results were statistically significant. The PASAT was the one subtest of the neuropsychological test battery which most sensitive to the positive effect of the Avonex®. Based on the PASAT results, there was a statistically significant performance improvement in information processing speed, memory, and new learning. In addition, the group treated with Avonex® slowed their progression of cognitive decline by 47% compared with placebo [44]. See Table 1.

The Betaseron® trials did not measure cognitive performance in the original trial. In a phase III trial, Pliskin et al. [90] studied 30 patients on medication and measured neuropsychological function. The neuropsychology battery included measures of immediate and delayed recall, visual reproduction, attention, mental speed, motor function, and depression. Improvement with treatment was seen in only 1 of 13 domains (i.e. visual memory) [90]. In a Cuban study, Interferon-alpha2b recombinant improved cognitive impairment in relapsing remitting MS. Subjects were treated and followed for 2 years and performance was measured on the Luria, WAIS, Benton, and PASAT-3. Treated subjects all improved and showed a dose response, with higher dose subjects performing significantly better than the low dose and placebo subjects [27]. In an Israeli study of interferon beta-1b, Barak and Achiron [15] showed that the treated group improved significantly on tests of complex attention and concentration, and visual learning and recall. There was no deterioration from baseline over the course of the 1 year study on verbal fluency, a measure which did worsen spontaneously along with the others mentioned above, in the non-treated group [15].

In a phase III trial with Copaxone® versus placebo, a brief repeatable battery of neuropsychological tests was administered at entry, at 12 and 24 months post-treatment. No significant trends were observed on any of the cognitive measures [121]. The mitoxantrone trials did not include cognitive measures in either their primary or secondary outcomes, though the drug did show a beneficial effect on disease progression measured by lesion accumulation on MRI [111].

Pharmacologic interventions to slow cognitive decline have also been tested in MS. A pilot, 12 week, open label trial of donepezil, in 2000, used the Mini Mental Status Examination to measure response to the drug in 17 patients with MS confined to a long term care facility. They did show statistically significant gains in attention, memory, and executive functioning. Greene et al. [97], in follow-up, performed a randomized trial of donepezil with 69 patients. Subjects received 10 mg per day of donepezil versus placebo, and at the end of twenty-four weeks there was demonstrated improvement on memory performance on the Selective Reminding Test, but no significant improvement on other tests of cognitive functioning. Memantine is a newer agent indicated in the treatment of moderately advanced Alzheimer's disease. Because of its efficacy and safety in dementia syndromes, it is used increasingly in patients with MS and cognitive impairment in an off-label fashion. No clinical trials have been published regarding the use of memantine in the treatment of cognitive impairment in MS, although there is one case report of its effectiveness in reducing pendular nystagmus in MS [68].

The clinical use of pharmacologic agents to enhance cognitive performance in MS is common, regrettably, without scientific evidence to support the practice. Adrenergic agents, such as methylphenidate and methylphenidate HCl, are used to combat mental fatigue, and improve processing speed as well as sustained attention. Dopaminergic agents, chiefly amanta-

Table 1
Three sets of composite variables used in the principle neuropsychological analysis of the Avonex multiple sclerosis trial

| Variable Set | Factor (Cognitive Domain) | Representative Variable |
|---|---|---|
| Set A<br>Information processing/Memory (frequently impaired in multiple sclerosis) | Information processing<br>Verbal Learning/recall<br>Visual Learning/recall | CALCAP Sequential RT<br>CVLT Trials 1–5 total<br>RFFT Error Ratio |
| Set B<br>Visuospatial abilities/Executive functions (Impaired often in multiple sclerosis) | Visual-spatial abilities<br>Visual Scanning<br>Problem Solving<br>Planning/sequencing<br>Deductive reasoning | WMS-R Visual span FWD<br>Visual # Search trials<br>WCST Perseverative responses<br>TOL Planning time (%)<br>20 Qs Good Hypothesis (%) |
| Set C<br>Verbal abilities/attention span (rarely impaired in multiple sclerosis) | General verbal abilities<br>Attention span | WAIS-R information (raw score)<br>WMS-R Digit span FWD |

Table 2
Summary of cognitive findings in Phase III immuno-modulating trials

| Agent | Dosage | N | Measures | Outcome |
|---|---|---|---|---|
| Interferon beta 1b (betaseron) | 8 Miu (250 $\mu$g) SC every other day | 30 | Immediate and delayed recall memory; attention/mental speed; DH and NDH motor function; depression | Significant improvement on 1 of 13 measures ($p < 0.003$) Weschler Memory Scale- Visual Reproduction Delayed Recall |
| Interferon beta 1a (Avonex®) | 30 $\mu$g IM once weekly | 166 | Information processing; learning memory (most often impaired in multiple sclerosis); visual spatial abilities; problem solving; verbal abilities; attention span | Significant improvement on information processing/learning memory ($p < 0.011$) slowed progression of cognitive deterioration by 47% compared with placebo based on the PASAT ($p = 0.02$) |
| Glatimer acetate (copaxone) | 20 mg SC once daily | 248 | Sustained attention and concentration; verbal learning and delayed recall; visual spatial learning and delayed recall; semantic retrieval | No effect on any of the NP tests |

[52] Galetta, Markowitz, Lee. Arch Intern Med 162;2002: 2161–2169.

dine, have been used with the intent of improving initiation and apathy. While the effect of 3-aminopyridine is favorable on the motor performance of patients with MS, no significant effect was demonstrated on cognitive performance in the single published study available for review [112]. Amantadine and pemoline were investigated for their effect in altering cognitive function in MS. These agents are commonly used for the fatigue associated with the disease. Neither drug was found to influence performance on tests of attention, verbal memory, nonverbal memory or motor speed. Cognitive function was concluded to be independent of fatigue by these investigators [55].

However, treating the associated symptoms of pain, sleep impairment, depression and fatigue intuitively seems to be of help in enhancing cognitive function. Despite the lack of published data to support that notion, these measures are commonly addressed in clinical practice. Correlations do exist between the effective treatment of depression and the improvement in fatigue; however those studies do not assess the effect of mood treatment on cognitive function [80,81]. Treatment of depression with serotonin reuptake inhibitors (SSRIs) is the standard of care in most clinical settings, and given the increased incidence of affective disorders in MS, this line of treatment should be a consideration in every MS patient who is receiving comprehensive disease management.

Consideration should also be given to those commonly used medications in MS which can negatively affect cognitive performance. Included are medications for spasticity, in particular, which are all potential central nervous system depressants. Tizanadine is likely to be the most sedating of the commonly used spasmolytic medications. Diazepam and clorazepate are effective antispasmodics but can worsen depression and slow cognitive activity. When given in small dose or frequent interval dosing (e.g. diazepam 2 mg q 4 hours) they are more tolerable and have less peak effect and thereby less sedation. The urologic medications given for spastic bladder are also central nervous system depressants to a degree given their anticholinergic action.

Newer agents such as oxybutynin chloride and tolterodine tartrate have fewer side effects in general than the short acting anticholinergic and spasmolytic agents and are more tolerable for patients with cognitive deficits or not. Finally, anticonvulsants are used frequently in MS to treat neuropathic pain. Some have more cognitive side effects than others, and lamotrigine appears to have the best cognitive side effect profile. Chronic use of valproic acid can lead to elevated ammonia levels and encephalopathy and levatiracetam has had reported "psychiatric" side effects, primarily in terms of paranoia. However, some patients have reported decreased concentration and new learning abilities while taking levatiracetam. Gabapentin is commonly used to treat both neuropathic pain and phasic spasm, but it can sedate patients and can negatively impact sustained attention.

## 8. Lessons learned from the treatment of cognitive disorders associated with other conditions

Empiric trials of medications for acquired cognitive disorders such as the dementia associated with traumatic brain injury or cerebrovascular disease have been considered for use in MS, based on the availability of those medications for off-label use and on their safety profile. Methylphenidate and other adrenergic compounds have been consistently used for the fatigue of MS in clinical practice, and there are anecdotal reports from patients receiving these therapies (e.g. methylphenidate, methylphenidate HCl, amphetamines) that their concentration improves, as does their speed of processing. There are no published clinical trials of those medications in MS, yet methylphenidate is in common use in the traumatic brain injury population. It is also used to combat the narcosis in chronic pain management. Dopaminergic agents have been commonly used in Parkinson's disease and improved initiation of motor bradykinesia. Their use has been extended to trials in nonfluent aphasias, and they have been used off-label as a method of arousal in the bradyphrenic acquired traumatic brain injury patient. Their use in MS has largely been confined to the treatment of fatigue (amantadine), but no reports as to the effectiveness of dopaminergic agents in treating the cognitive impairment of MS exist. Interestingly, selegiline (a monoamine oxidase B inhibitor) was shown to improve verbal memory in HIV dementia [7].

The cognitive deficits seen in Parkinson's disease are similar in several ways to those seen with MS. For example, patients with Parkinson's disease also fail in areas of executive function, planning, initiation, and monitoring of goal directed behaviors. Dopaminergic agents may enhance cognition in some patients with Parkinson's disease and not in others. This may be a function of the level of dopamine depletion in certain areas of the brain relative to others, specifically the basal ganglia and prefrontal cortex [67] and requires further study.

Modafinil has been effectively used as an antidote for the fatigue encountered in MS. Its effectiveness in enhancing speed of processing, sustained attention, or new learning has not been directly examined. The initial FDA approved use was to combat daytime drowsiness associated with narcolepsy. Those original trials in assessing efficacy in fatigue did not include any cognitive measures as part of the outcome data [93]. However, a 2005 study in Neurology casts doubt on the effectiveness of modafinil in treating MS-related fatigue, as measured by the modified Fatigue Impact Scale [115].

## 9. Non-pharmacologic treatments for cognitive impairment in MS

The goals of non-pharmacologic treatment for cognitive impairment in MS are similar to the goals for immuno-modulating drugs. They involve preventing progression of cognitive disability, promoting a therapeutic "milieu" in which optimal cognitive functioning can occur and specific interventions which are known to be effective in remediating cognitive disorders of any etiology. Behavioral and life style changes can make a significant impact on a person's functioning. These might involve strategies as simple as avoiding cigarettes (known to increase the risk of transforming relapsing remitting into secondarily progressive MS) [60] or increasing the amount of restful sleep. The effect of regular, moderate exercise enhances pain tolerance, improves sleep and appetite, and mitigates spasticity. It also improves fatiguability, but it remains to be seen if it tends to allow greater concentration or sustained attention to task and reduced distractibility. Specific regimes for exercise in MS have never been validated and general principles of healthy exercise prevail. Both cardiopulmonary and musculoskeletal conditioning should be addressed where possible through a balanced program of aerobic and resistance training.

Yoga and aerobic exercise were shown to improve fatigue, but not performance, on measures of attention and alertness [84].

While interest in dietary supplements such as folic acid and magnesium is growing, there are no dietary guidelines specific for MS. Patients with MS are encouraged to eat a balanced diet, and avoid excessive intake of processed foods, alcohol, and stimulants such as caffeine and chocolate. Mega-dosing vitamins is not recommended, particularly fat-soluble ones, and patients are encouraged to take vitamin C 1000 mg/day, calcium 1000–1500 mg/day, vitamin D 400 units per day, folic acid 1–2 mg/day and a multivitamin. Many patients also consume 800 IU of vitamin E daily, as well. Recent studies pointing to the prevention of cisplatin-induced neuropathy with the addition of vitamin E to dietary intake [12] and the slowing of Alzheimer's disease progression [40] with the use of folic acid, make these recommendations reasonable leaps from evidence in other disease states. Statins are not routinely prescribed for cognitive impairment or relapse control because of lack of scientific and clinical care data, and because of the potential for adverse events, such as myalgia, myositis, and liver dysfunction.

## 10. Cognitive rehabilitation in MS

Cognitive rehabilitation (CR) is a treatment that has been incorporated into several clinical fields, such as occupational therapy, neuropsychology, and nursing. Sohlberg and Mateer [113] have stated a preference for the term "rehabilitation of individuals with cognitive impairment" rather than "cognitive rehabilitation" because they feel the former term better describes a patient-focused endeavor. Cognitive rehabilitation may be defined as a system of interventions designed to increase daily functional abilities by means of two general approaches: ameliorating a patient's deficits in processing and interpreting information (restorative) and modifying the patient's environment (compensatory). Cognitive rehabilitation programs often utilize a combination of these approaches, with functional improvement as the desired outcome. The restorative approach depends on the possibility that the brain is capable of cortical reorganization following injury (i.e. that the brain possesses some degree of plasticity) [116]. Some authors have expressed disappointment that restorative CR has rarely been found to have significant impact on everyday functioning [73]. Ideally, CR programs are individualized to meet patients' specific needs and goals.

Complex interrelationships among cognitive domains may increase the difficulty of designing optimal, individualized CR programs for patients. For example, it may be difficult to disentangle the relative contributions of attention, working memory, and executive functions to a patient's cognitive difficulties. This difficulty ensues from the intricate circuitry connecting neuroanatomical substrates of these cognitive domains.

Several exhaustive reviews of existing research related to the efficacy of CR among patients with TBI or stroke have been published in the last five years. These reviews have found evidence supporting the efficacy of specific CR interventions for improving attention, memory, and executive function deficits [1,28]. The researchers also reported support for the use of compensatory devices to augment memory. Another evidence-based review of the literature related to the efficacy of CR with TBI and stroke patients was compiled by a committee representing a section of the American Congress of Rehabilitation Medicine. Overall, the authors found support for the effectiveness of several forms of CR. They recommended specific forms of CR for the remediation of language deficits following left hemisphere stroke, perceptual deficits following right hemisphere stroke, and deficits of attention, memory, functional communication, and executive functioning following TBI [32].

Multidisciplinary rehabilitation specifically directed at cognitive and physical remediation has been shown to be effective in relation to disability, quality of life, and emotional well-being in MS [51]. However, compared to the TBI and stroke literatures, the number of investigations into the efficacy and effectiveness of treatments for cognitive impairment in MS is low. The progressive nature of MS may have discouraged some researchers and clinicians from addressing the topic [73]. Of the few studies that have addressed CR in MS most did not find evidence for significant improvement in cognitive functioning following treatment. One study involved two groups of 20 moderately disabled MS patients who were receiving physical therapy. The treatment group received CR and psychotherapy, while the controls received sham "mental stimulation." Results indicated the only significant improvements in the performance of the treatment group, as compared to the controls, were lower Beck Depression Inventory (BDI) [20] scores and increased visual perception abilities [63]. No other effects were seen in cognitive domains.

Another study of group-format outpatient treatment among 27 MS patients randomly assigned to either treatment or control group reported significant improvements in word-list learning and "abstraction" among the treatment group as compared to controls. However, the nature of the cognitive treatment in this study is unclear; treatment sessions were reportedly comprised of "physical and mental exercises," alongside art, music, meditation, relaxation, and other components [113]. Finally, 14 MS outpatients underwent extensive CR treatment; participants attended 17 weeks of treatment, consisting of two 90-minute CR sessions and one 50-minute stress-management session per week. No significant improvement in cognitive functioning following treatment was found [47]. Other more recent studies of cognitive interventions in the treatment of MS have similarly resulted in non-significant findings [74,114]. The elements of cognitive training were not defined or standardized in any of the above studies, so it is unknown if task-specific training would have a more significant impact on measures of functioning. A systematic program of CR created and maintained improvement in performance on specific cognitive and functional tasks in mildly impaired AD patients [76], therefore it might be possible to achieve similar results when task-specific programs are directed at cognitively impaired persons with MS.

Many studies in MS have sought to train a specific skill, but it is unclear if that strategy translates into global changes in function, such as the ability to continue to dwell independently in the community, or maintain employment status. Twelve-week training sessions designed to reduce egocentric speech improved appropriate social behavior, but no outcome measures were directed at global functioning, and sustained effect of the treatment was not reported [23]. Computer-aided retraining of memory and attention was not found to be useful in a group of MS patients, although both the training and control groups received computer-aided training. The treatment group received training in memory and attention, and the control group received training in visual motor coordination. Both groups improved on brief neuropsychological screens assessing memory and attention, therefore, it was concluded that specific attention and memory training is not efficacious [114]. However, it could be argued that just about any type of training may be efficacious in improving mental alertness and speed of processing in the cognitive impairment of MS, though this study was unable to draw that conclusion. Repetition alone is insufficient to compensate for new learning impairments among persons with MS and more intense cognitive training strategies may be required than just rehearsal alone [31].

Group and individual therapies for support and coping have improved the fatigue of patients with MS, and patients report improvements in "vitality" and fatigue, which could positively influence cognitive function, though neuropsychological measures were not assessed in these studies [80,117]. Energy conservation techniques have successfully been used by and taught to patients, with the end result being that patients with MS are able to accomplish more, improve their performance, and prioritize effectively. This type of intervention is cognitively based, as it is a method of teaching strategies and should be viewed as a form of cognitive remediation [77,124]. No direct effect was seen after similar treatment approaches in a study by Jonsson on cognitive measures, but the depression inventories appeared to have improved substantially [63].

There was no comment on change in real life function in any of these studies, and it is not entirely clear how or if changes in mood or on neuropsychological profiles truly reflect enhancement in real life function. It is implied that they do, but the correlation is not direct. A similar impression was gained when MS patients in a residential home were treated cognitively via the use of memory books. No changes in neuropsychological measures were seen, but improvement in mood was documented [78].

Despite these generally inconclusive findings, a few recent studies have suggested ways of successfully incorporating CR into treatment of MS patients. Scarrabelotti and Carroll found that implicit memory (i.e. automatic or unconscious memory), is preserved in MS. In contrast, the authors point out that consciously controlled information processing is often impaired [107]. Therefore, the authors suggest that a CR program incorporating automatic, unconscious memory rather than conscious memory may enable MS patients to develop improved memory abilities. For example, a patient could be instructed to notice an aspect of a stimulus (e.g. letters or numbers placed in a grid), but not instructed to try to remember the stimulus. The authors note that the appropriateness of applying this strategy would depend on the properties of the stimulus (e.g. complexity).

Additionally, the authors of a study on the impact of information processing speed deficits on cognitive performance among MS patients offered a different strategy for incorporating CR. The primary finding was that working memory deficits in their sample of MS patients was due to impaired speed, rather than decreased accuracy, of performance. The authors assert that offering additional time to MS patients for performance of a memory-based task may result in strengthening "the quality of encoding of to-be-learned information" [37–39].

## 11. Summary

Recognition and typing of cognitive deficit in MS have advanced substantially over the last 15 years, but the precise combination of medications and behavioral training thought to prevent or slow its progression remain uncertain. Equally unclear is our ability to predict which subsets of persons with MS will be afflicted with cognitive impairment and to what degree. While it may be intuitive to link effective treatment of fatigue with improvement in cognitive performance, available research has yet to bear that out. Specific task training techniques do not clearly generalize well in patients with MS. Emotional well-being and stabilization of affective disorders does positively affect cognitive performance. Complementary therapies and exercise remain unproven in their ability to enhance cognitive performance. Cognitive remediation must be comprehensive, probably best done in a real world context, and individualized. These tailor-made programs must account for stage and type of disease as well as psycho-social problems specific to the patient with MS [92].
